# Portal Hypertensive Duodenopathy Manifesting as “Kissing” Duodenal Ulcers in a Nigerian with Alcoholic Cirrhosis: A Case Report and Brief Review of the Literature

**DOI:** 10.1155/2012/618729

**Published:** 2012-10-21

**Authors:** Aderemi Oluyemi, Adeniyi Amole

**Affiliations:** ^1^Department of Medicine, General Hospital, Ikorodu, Lagos State, Nigeria; ^2^Lister Medical Center, Ogba, Lagos State, Nigeria

## Abstract

Multiple duodenal ulcers are an uncommon finding in portal hypertensive duodenopathy (PHD). They represent a potential source of clinically significant bleeding from the upper gastrointestinal system in patients with cirrhosis. As this particular ulcer entity in relation to PHD has no distinguishing symptoms aside from those relating to the consequent bleeding, most of them are found either on routine endoscopic screening for cirrhotics or on endoscopic examination for cause(s) of bleeding in this patient population. The case documented below highlights many of the aspects of pathogenesis, associations, and consequences of this unique endoscopic finding in cirrhotic patients.

## 1. Introduction

Portal hypertensive duodenopathy is a known association of portal hypertension. It is clinically significant as it has potential for being a source of upper gastrointestinal bleeding. Hence, it could bear important consequences on mortality and morbidity in this condition.

Scientific literature documents that multiple ulcerations in the duodenum is a finding in this condition. But this finding is uncommon. We detail a case of a 42-year-old Nigerian man with alcohol-related decompensated cirrhotic liver disease who had previously undergone multiple endoscopic variceal ligation sessions for esophageal varices who now presents with features of repeated upper gastrointestinal bleeding and was found to have two distinct ulcers in his duodenal cap—“kissing ulcers”.

## 2. Case Report

A 42-year-old clerk presented with a history of progressive weakness and an episode of loss of consciousness. Thirteen months earlier, the gentleman had been diagnosed with alcohol-related decompensated chronic liver disease but had not adhered to the prescribed abstinence from alcohol.

About two months prior to presentation, he was diagnosed with grade 3 esophageal varices which had required 2 sessions of endoscopic band ligation (EBL)—each session was one month apart from the other and the latest EBL session was about four weeks prior to this presentation. Presently, the patient reported that he had become increasingly weak after the initial improvement he noted following the second EBL session, his stools had become dark and tarry, and that he had an episode of loss of consciousness which lasted a few seconds. He denied using steroid or nonsteroidal anti-inflammatory drugs. Following the last EBL session, his weekly packed cell volume values were: 29%, 28%, 29%, and 22%-the last one was done 12 hours before presentation. Other laboratory findings put him in Child-Pugh class B.

The vital signs revealed a pulse rate of 124 beats per minute which was low volume and his blood pressure was 98/64 mmHg. His examination findings were significant for dyspnea at rest (respiratory rate was 28 cycles per minute), pallor, and the digital rectal examination revealed black stools.

**Figure 1:**
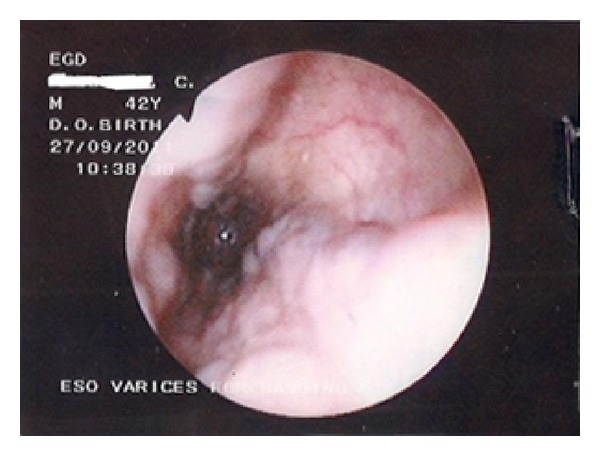
Endoscopy of esophagus showing the grade 1 (enlarged but straight) varices.

**Figure 2:**
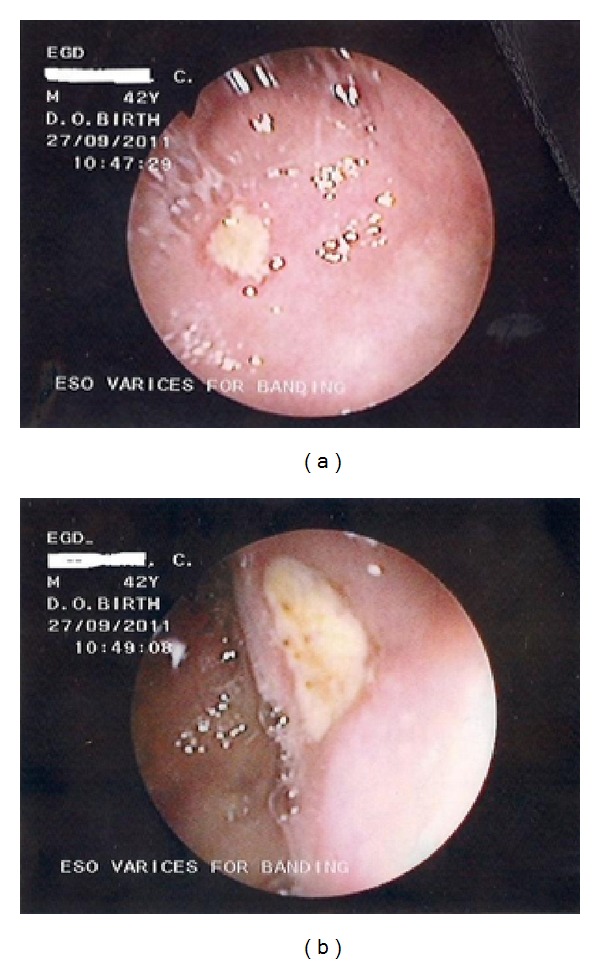
(a) Endoscopic view of the duodenum showing the ulcer on its anterior wall. (b) Endoscopic view of duodenum showing the ulcer on its posterior wall.

After stabilization, he underwent an esophagogastroduodenoscopy (EGD) which revealed enlarged but straight varices (grade 1) (Figure 1). No features of PHG were noted. The duodenal mucosal lining was erythematous and edematous. There were two duodenal ulcers noted on the walls in the first part—“kissing ulcers” (Figures 2(a) and 2(b)). The ulcer on the anterior wall was about 5 mm by 4 mm while the one on the posterior wall was smaller- 2 mm by 3 mm. They were both circular and their bases were filled with whitish exudates, while the larger of the two ulcers had evidence of recent bleeding in the presence of multiple blood clots at its base. The histopathology report of the biopsies taken showed subepithelial edema and dilation of mucosal/submucosal capillary vessels with strikingly minimal inflammatory changes. *Helicobacter pylori* could not be demonstrated.

The session was completed with EBL for the varices seen and the patient was discharged in stable clinical condition. Medical management of the condition was reinstituted and he was referred for a liver transplant. He was lost to followup shortly thereafter.

## 3. Discussion

Portal hypertension (PH) is known to be associated with the development of mucosal changes in the gastrointestinal tract (GIT)—the so called “congestive gastroenteropathy” [1] or “portal hypertensive syndrome” [2]. By far the most dreaded of these changes is the development of esophageal varices with their clinically devastating consequence of GIT bleeding. McCormack et al. in 1985 [3] gave a detailed pathological description of gastric mucosal abnormalities associated with PH. Thereafter, it has been shown that PH changes can affect all parts of the GIT and the entities have since acquired names according to the regions involved—portal hypertensive gastropathy (PHG) [3], duodenopathy (PHD) [4], enteropathy (PHE) [5–7], and colonopathy (PHC) [8].

A consensus definition of PHD is not available at this time but various workers have considered many endoscopic and histological features to be consistent with a diagnosis of the disease. These endoscopic findings can be classified after Barakat et al. [9] as (a) mucosal erythema (patchy or diffuse), (b) mucosal edema, (c) mucosal breaks (erosions or ulcers), and (d) vascular lesions (varices or telangiectasia). Other rare lesions such as duodenal polyps have also been reported [10]. Vascular changes dominate as the main histologic feature characterizing this portal congestive process—they include both capillary congestion/dilatation and capillary angiogenesis [9, 11]. Along with capillary changes are fibrous proliferation and increased apoptosis, all in a background of absent/minimal inflammatory cells. The presence or otherwise of villous atrophy is still controversial [12]. Interestingly, it has been shown that endoscopically normal duodenal mucosa does not preclude the histological changes of PHD [9].

The rare finding of multiple duodenal ulcers in cirrhotics has only been documented in a handful of published works [9]. The endoscopic findings in this index case represent, to the best of authors' knowledge, the first time this is being documented from our local environment. The histological report from the index patient was most consistent with the endoscopist's diagnosis of PHD.

The clinical importance of PHD derives from the fact that it is a recognized cause of occult or overt bleeding. Bleeding is more commonly related to erosions and/or ulcers [9] but erythematous duodenopathy [1] and even polyps [10] have been reported to cause bleeding as well. Though bleeding can be severe and require intervention [10, 13, 14], fortunately, most episodes of overt bleeding are self-limited [9]. Our patient's case highlights this fact as his was severe bleeding with associated cardiovascular compromise and yet at had become quiescent at EGD and required no further intervention.

Ever since the awareness of the disease had been created, several attempts have been made to correlate the presence and severity of PHD to various factors such as severity and etiology of liver disease, manifestations of PH in other sites of the gastrointestinal tract, a history of upper gastrointestinal bleed, anemia, and so forth, [2, 15]. Interestingly, there has not been shown any significant relationship between the presence nor severity of PHD lesions with the severity nor etiology of liver disease [2, 15, 16]. In the index case, grade 1 esophageal varices were seen along with the double duodenal ulcers while these were absent when he had a worse grade of esophageal varices. His Child-Pugh (CP) score had also been downgraded with prior instituted management. He was CP class B as at presentation and this was the first time that PHD was being noted.

Data concerning the relationship of presence and severity of PHD with previous attempts at esophageal variceal eradication has been conflicting-scientific literature bearing studies that both support [2, 17] and refute it [5, 15, 18]. But a 2010 work from Egypt represents the most detailed and specifically designed prospective study that examined this puzzling question [19]. The results were in the affirmative as the paper showed that PHE changes (PHD included) increased in frequency and severity after esophageal variceal obliteration. The case detailed here bears witness to this as our patient showed no PHD features till he had had 2 prior sessions of EBL.

## 4. Conclusion

This report highlights the fact that multiple duodenal ulcerations are a feature of PHD and further underscores the need for adherence to international standards for the care of all cirrhotics by carrying out regular periodic EGDs to access for such mucosal abnormalities as this. The authors note that this is easier said than done in a resource limited environment like ours on account of EGD-related limitations in availability, affordability, accessibility, and scarcity of relevant expertise. We also note the usefulness of intubation of the duodenum as a relevant and necessary component of EGD particularly in patients with liver disease—this becomes even more pressing in those that have undergone interventions for PHD-related lesions.
